# Biannual Mass Azithromycin Distributions for Preschool Children and Malaria Parasitemia: A Secondary Analysis of the MORDOR Cluster Randomized Trial

**DOI:** 10.1001/jamanetworkopen.2025.27148

**Published:** 2025-08-18

**Authors:** Ahmed M. Arzika, Amza Abdou, Ramatou Maliki, Elodie Lebas, Catherine Cook, Benjamin Vanderschelden, Kieran S. O’Brien, Sun Y. Cotter, Nicole E. Varnado, E. Kelly Callahan, Robin L. Bailey, Sheila K. West, Philip J. Rosenthal, Travis C. Porco, Thomas M. Lietman, Jeremy D. Keenan

**Affiliations:** 1 The Carter Center, Niamey, Niger; 2 Centre de Recherche et Interventions en Santé Publique, Birni N’Gaoure, Niger; 3 Programme Nationale de Santé Oculaire, Niamey, Niger; 4 Francis I. Proctor Foundation, University of California, San Francisco; 5 Department of Ophthalmology, University of California, San Francisco; 6 Department of Epidemiology & Biostatistics, University of California, San Francisco; 7 The Carter Center, Atlanta, Georgia; 8 London School of Hygiene and Tropical Medicine, London, United Kingdom; 9 Dana Center for Preventive Ophthalmology, Johns Hopkins University, Baltimore, Maryland; 10 Department of Medicine, University of California, San Francisco; 11 Institute for Global Health Sciences, University of California, San Francisco

## Abstract

**Importance:**

Mass azithromycin distributions may reduce malaria parasitemia in the short term, but longer-term effectiveness is unclear.

**Objective:**

To examine whether biannual mass azithromycin distributions are associated with lower rates of malaria parasitemia in preschool children living in Niger.

**Design, Setting, and Participants:**

A cluster randomized trial was performed from November 23, 2014, until June 9, 2020, as an ancillary trial to a larger trial studying the effect of mass azithromycin on child mortality. Study communities (ie, government-defined health catchment areas) in Niger were randomized in a 1:1 ratio to biannual (ie, twice-yearly) mass administration of azithromycin or placebo to all children aged 1 to 59 months and followed up for 5 years. Data analyses were performed from June 25, 2023, to April 27, 2025.

**Intervention:**

Twice-yearly administration of a single dose of oral azithromycin, 20 mg/kg, or placebo.

**Main Outcomes and Measures:**

The prevalence of parasitemia 4 years after the community started treatment, assessed in a random sample of 40 children per community.

**Results:**

Among the 30 communities in Niger included in the study at baseline, the 15 communities randomized to azithromycin consisted of 1695 children (mean [SD] age, 30.8 [2.8] months; 858 [51.8%] male) and the 15 communities randomized to placebo consisted of 3031 children (mean [SD] age, 30.6 [2.6] months; 157 [52.0%] male). The mean prevalence of malaria parasitemia at baseline was 8.9% (95% CI, 5.1%-15.7%) in the azithromycin arm and 6.7% (95% CI, 4.0%-12.6%) in the placebo arm. At annual follow-up visits up until month 48, parasitemia was not statistically significantly lower in the azithromycin arm compared with the placebo arm, assuming a 10% prevalence in the placebo arm (−3.3 percentage points [PP]; 95% CI, −5.8 to −0.2 PP; permutation *P* = .05). The Niger Ministry of Health instituted seasonal malaria chemoprevention (SMC) after the month 36 study visit. Analysis restricted to the period before SMC found significantly less parasitemia in the azithromycin arm compared with the placebo arm (4.8 PP lower; 95% CI, −7.4 to −1.3 PP; permutation *P* = .02).

**Conclusions and Relevance:**

In this placebo-controlled cluster randomized trial, malaria among children aged 1 to 59 months was lower in communities treated with biannual mass azithromycin, but the effect was significant only for the first 3 years of the trial, before SMC.

**Trial Registration:**

ClinicalTrials.gov Identifier: NCT02048007

## Key Points

**Question:**

Do communities in rural Niger treated with mass azithromycin distributions have lower rates of malaria parasitemia among children aged 1 to 59 months compared with untreated communities?

**Findings:**

In a cluster-randomized trial that enrolled 4726 children aged 1 to 59 months at baseline from 30 communities in Niger, communities treated with 5 years of twice-yearly mass azithromycin distributions had a lower prevalence of malaria parasitemia compared with communities treated with placebo. However, the difference was significant only for the initial 3 years of the study, before the institution of a seasonal malaria chemoprevention program that provided monthly mass antibiotics during the malaria season.

**Meaning:**

These findings suggest that biannual mass azithromycin distributions were associated with a significant reduction in malaria parasitemia before, but not after, the institution of seasonal malaria chemoprevention.

## Teaser

This cluster randomized trial of preschool children living in Niger examines the association of biannual mass azithromycin distributions with lower rates of malaria parasitemia.

## Introduction

The World Health Organization currently recommends mass drug administration (MDA) of azithromycin for children aged 1 to 11 months in Sub-Saharan African settings with high child mortality in an attempt to improve child survival.^1^ This recommendation is based on the results of randomized trials that have found mass azithromycin distributions to reduce mortality among preschool children.^2,3,4,5^ The mechanism by which azithromycin MDAs reduce child mortality is not known, but azithromycin is a broad-spectrum antibiotic with activity against many pathogens, including malaria.^6,7,8,9,10,11,12^

The Macrolides Oraux pour Réduire les Décés avec un Oeil sur la Resistance (MORDOR) trial was a cluster randomized trial performed in Malawi, Niger, and Tanzania in which communities were assigned to biannual MDA with a single dose of azithromycin or placebo, targeted to children aged 1 to 59 months.^3^ The trial was cluster randomized to account for potential direct effects of azithromycin (ie, to clear infection in a treated individual) as well as indirect effects (ie, to prevent transmission to others). MORDOR found a 14% reduction in childhood mortality in communities randomized to 2 years of biannual mass azithromycin distributions. Malaria was a major cause of death in each of the 3 sites, and mass azithromycin led to a significant reduction in malaria parasitemia at 24 months in an ancillary trial at the Niger site, although not at the Tanzania or Malawi site.^11,13,14^ The azithromycin and placebo interventions were continued for an additional 3 years at the Niger site, providing longer-term data on the impact of mass azithromycin on malaria outcomes. We hypothesized that the prevalence of malaria parasitemia would remain lower in the azithromycin arm during the study.

## Methods

### Study Design

**Figure 1.**
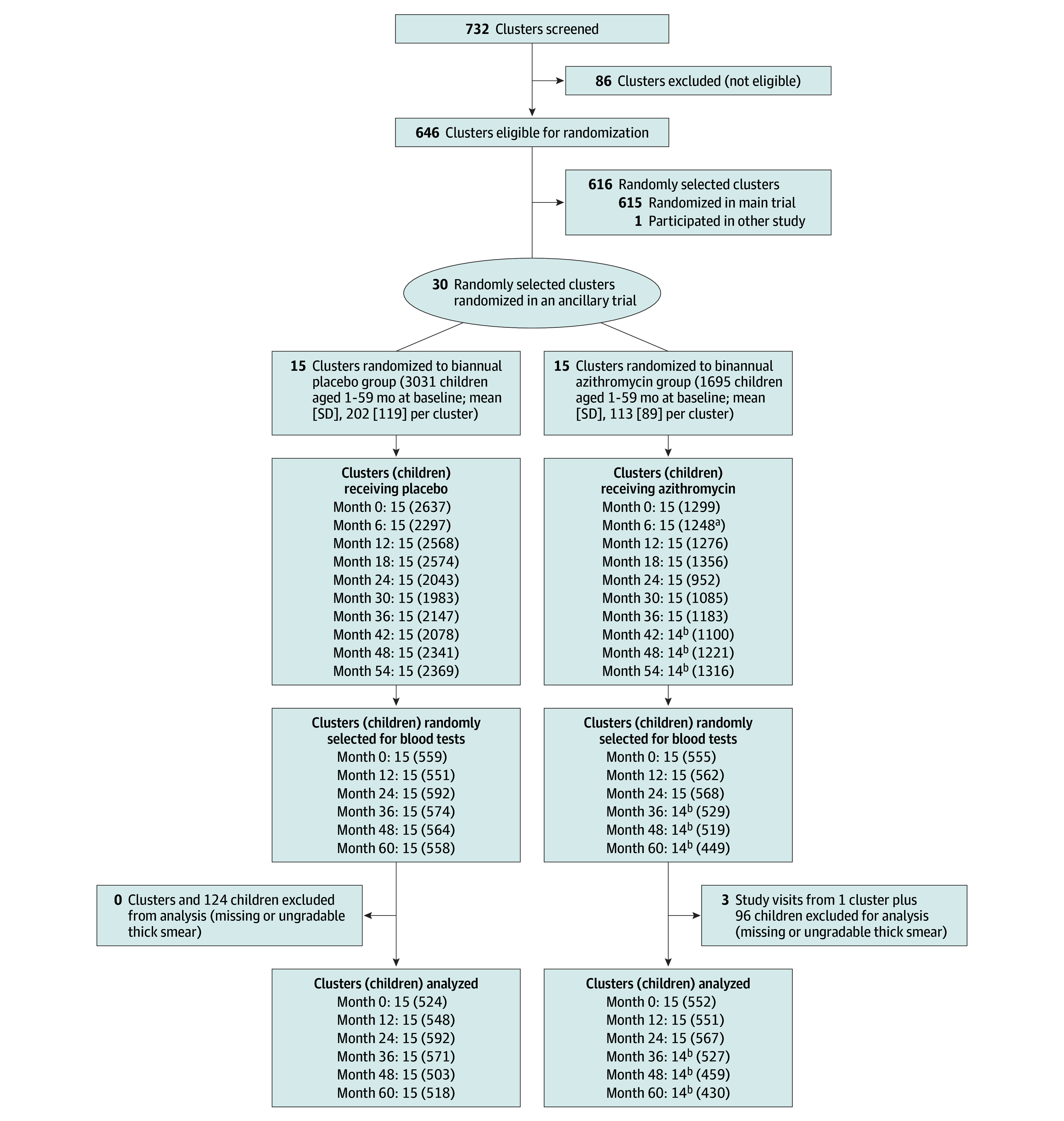
Trial Flow Communities in the study area with a population of 200 to 2000 were eligible and randomized to this trial (n = 30), the main MORDOR (Macrolides Oraux pour Réduire les Décés avec un Oeil sur la Resistance) trial (n = 615), or an ancillary trial on antibiotic resistance (n = 1). A door-to-door census was performed approximately every 6 months, at which time the study drug was administered. Cross-sectional random samples of children from each community were monitored every 12 months for malaria outcomes. Children excluded from analysis had thick smears that were missing or unreadable. ^a^Does not include data from one entire community that was known to be treated but had missing antibiotic data due to technical problems. ^b^One community declined to participate in monitoring visits after month 24 and declined to receive antibiotics after month 36.

A parallel-group cluster randomized trial was performed in 30 communities of the Boboye and Loga departments of Niger as an ancillary trial accompanying the main MORDOR trial (Figure 1). The 30 communities were randomly drawn from the same pool of communities as the main MORDOR trial and subsequently randomized in a 1:1 ratio to the same interventions as the main trial: biannual (ie, every 6 months) mass distribution of azithromycin or biannual mass distribution of placebo to all children aged 1 to 59 months (eFigure 1 in Supplement 1). Malaria outcomes were assessed annually in a random sample of children from each community. The study was designed as a 2-year trial that would be continued for an additional 3 years if the main trial found a significant reduction in mortality in the azithromycin arm. Communities in the ancillary trial were treated as originally allocated for the entire 5-year study period. Data collection was performed from November 23, 2014, to June 9, 2020. Ethical approval for the main trial and ancillary trial was obtained from the Committee on Human Research at the University of California, San Francisco and the institutional review board of the Niger Ministry of Health. Guardians of children provided oral informed consent for both treatment and monitoring. The trial protocol is available in Supplement 2. This report follows the Consolidated Standards of Reporting Trials (CONSORT) reporting guideline.

### Participants

The unit of randomization for the trial was the *grappe*, a government-defined health catchment area. Grappes, hereafter referred to as communities, with a population between 200 and 2000 on the 2012 government census were eligible for the study. All children aged 1 to 59 months weighing 3800 g or more were eligible for treatment. A random sample of 40 children per community aged 1 to 59 months was monitored each year for malaria (ie, repeated cross-sectional random sampling). Seasonal malaria chemoprevention started in the study area in July 2018 (ie, approximately 2 months after the 36-month visit) and consisted of monthly mass distribution of a single dose of sulfadoxine-pyrimethamine plus a 3-day course of amodiaquine to children aged 3 to 59 months during a 4-month period of high malaria transmission (July to October) each year.^15^

### Randomization and Masking

The trial biostatistician (T.C.P.) randomly allocated communities in equal proportions to 1 of 6 letters, with 3 coded for azithromycin and 3 coded for placebo (sample function in R). The drug manufacturer (Pfizer Inc) provided the azithromycin and placebo and labeled each bottle with 1 of the 6 treatment letters. The Nigerien study coordinator enrolled communities and assigned the allocated intervention. Allocation was concealed at the cluster level by enrolling all communities before randomization and at the individual level by administering the treatment to all eligible children. Study bottles, packaging, and the appearance of the drug and placebo were identical. Participants and their guardians, field and laboratory staff, and all investigators except the trial biostatistician were masked to treatment allocation.

### Census and Monitoring

A door-to-door census was performed approximately every 6 months to enumerate all children 12 years and younger. Methods have been described previously.^3^

Malaria outcomes were assessed during the spring months each year, prior to the major seasonal malaria peak. Aside from the baseline visit, communities received azithromycin distributions approximately 6 and 12 months prior to the monitoring visit. Using blood from a finger stick, hemoglobin was measured (Hb 201^+^ device [Hemocue]), and then a thick and thin smear were prepared on a single slide. A random-number label was attached to each slide to mask the laboratory staff. The thin smear was fixed with methanol in the field. For ethical reasons a rapid diagnostic test for malaria was performed if the local study staff suspected the child of having malaria or if the child had a fever; those testing positive were referred to the local government health facility. Laboratory work was performed at the government clinical laboratories of Boboye and Loga, the Centre Hospitalier Regional de Dosso, the Hospital National de Niamey, and the Centre de Recherche Médicale et Sanitaire (Niamey, Niger). After staining slides with 3% Giemsa, 2 independent experienced laboratory workers masked to treatment allocation assessed thick smears for the presence and density of parasites and gametocytes and thin smears for malaria species using methods recommended by the World Health Organization.^16^ Discrepancies were adjudicated by a third independent laboratory worker.

### Intervention

Study drug was administered during a door-to-door campaign after the annual monitoring visit or as part of the census for the intervening study periods. Typically, MDAs occurred in the spring and fall, although with some variability from year to year (eFigure 2 in Supplement 1). A single directly observed dose of azithromycin suspension (20 mg/kg, calculated by weight for small children and approximated by height for children who could stand) or the same volume of identical-appearing placebo was administered.^17^ All children aged 1 to 59 months enumerated at the most recent census were offered treatment. Guardians were instructed to report any adverse events to a village representative, who communicated this information to the study coordinator.

### Outcomes

The prespecified primary malaria outcome was the prevalence of parasitemia in children aged 1 to 59 months in each community, as assessed from thick blood smear. Prespecified secondary outcomes included parasite density, gametocyte density, hemoglobin concentration at the individual level, and prevalence of anemia (hemoglobin <11 g/dL [to convert to grams per liter, multiply by 10]) at the community level.^18^

### Statistical Analysis

Community-level prevalence estimates for postrandomization monitoring visits were modeled in mixed-effects linear regression models with terms for treatment arm, study visit, and baseline prevalence, a random intercept for community, and an unstructured covariance structure. Prevalence outcomes were square root transformed because doing so improved the normality and heteroscedasticity of residuals; as a result, the magnitude of effect was not linear, and for ease of interpretation was expressed relative to a 10% prevalence in the placebo arm. The prespecified primary analysis included data up until the month 48 study visit; additional nonprespecified analyses were performed at month 36 (ie, the period before SMC started) and month 60 (ie, the final study visit). Intraclass correlation coefficients were derived from the regression models at the level of the randomization unit. Individual-level analyses for the parasite density and hemoglobin outcomes were modeled in similar models but not adjusted for baseline values. Analyses of parasite density were log-transformed.

On the basis of previous work in Niger,^12^ we anticipated a prevalence of malaria parasitemia in untreated communities of 25% and an intraclass correlation coefficient of 0.056. Assuming a 2-sided α = .05, enrolling 40 children in each of 15 communities per arm would provide more than 80% power to detect a 13% absolute difference between the 2 treatment arms.

*P* values were determined by Monte Carlo permutation (10 000 permutations), with a 2-sided significance level of *P* < .05 for the main analysis and no statistical adjustment for the prior analysis at 24 months given the timing of the contingency trial.^13^ Other analyses were deemed exploratory and had a 2-sided significance level of *P* < .05. Analyses were performed in an intention-to-treat fashion using R software, version 4 (R Project for Statistical Computing) and Stata, version 17 (StataCorp). Data analyses were performed from June 25, 2023, to April 27, 2025.

## Results

There were a total of 30 communities in Niger included in the study. At baseline the 15 communities randomized to azithromycin consisted of 1695 children (mean [SD] age, 30.8 [2.8] months; 858 [51.8%] male and 817 [48.2%] female) and the 15 communities randomized to placebo consisted of 3031 children (mean [SD] age, 30.6 [2.6] months; 157 [52.0%] male and 1455 [48.0%] female). Baseline characteristics were similar in the 2 arms except that communities in the placebo group were on average larger than those in the azithromycin group (Table 1). All communities received their allocated study medication and remained in follow-up, except for a single community in the azithromycin arm that declined monitoring visits after month 24 and declined treatments after month 36 due to community leader unwillingness to permit monitoring visits. The baseline characteristics of the study community lost to follow-up were similar to the other communities (eTable 1 in Supplement 1). Study drug was distributed to 85.3% (95% CI, 78.7%-91.6%) of eligible children in the azithromycin group and 87.7% (95% CI, 82.0%-93.0%) of eligible children in the placebo group across the 10 study visits (Figure 1). The timing of monitoring visits, treatment, and the rainy season was similar between the treatment arms (eTable 2 in Supplement 1). No hospitalizations or life-threatening illnesses were reported in either study group.

**Table 1.** Baseline Characteristics of the Study Communities as Assessed From a Population Census

Characteristic	Mean (SD)	
	Placebo (n = 15)	Azithromycin (n = 15)
Children aged 1-59 mo, No.	202 (113)	113 (89)
Sex, %		
Male	52.0 (4.2)	51.8 (4.7)
Female	48.0 (4.2)	48.2 (4.7)
Age, mo	30.6 (2.6)	30.8 (2.8)
Age group, y, %		
0	14.6 (4.8)	13.8 (4.6)
1	14.4 (3.9)	15.2 (5.1)
2	19.3 (3.3)	18.8 (5.0)
3	23.8 (4.4)	24.6 (5.3)
4	27.9 (6.9)	27.5 (5.8)
Female head of household, %	1.5 (1.0)	1.7 (1.0)
No. of children aged 1-59 mo per household	2.3 (0.5)	2.3 (0.7)
Household elevation, m	203 (26)	210 (27)
Distance from household to main road, km	6.5 (6.6)	6.6 (5.2)
Distance from household to town, km	39.3 (22.7)	33.6 (18.7)

**Figure 2.**
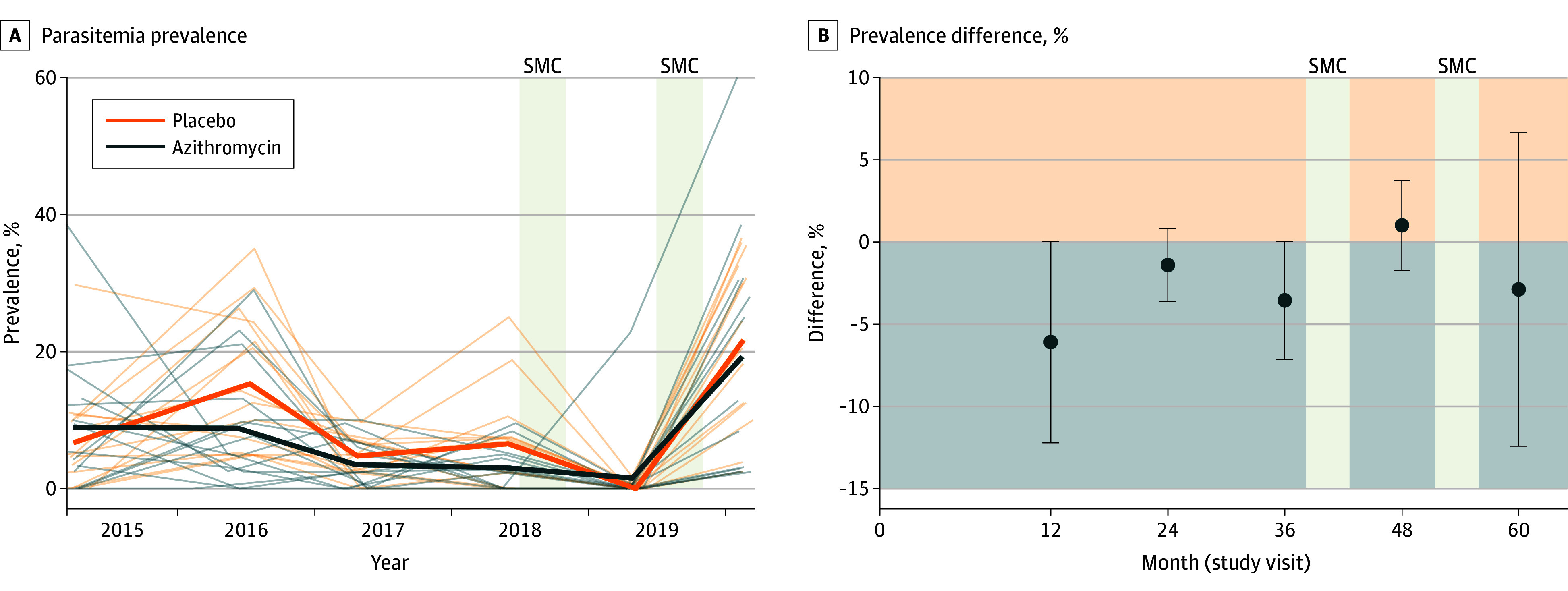
Malaria Parasitemia Among Children Aged 1 to 59 Months A, Thin lines represent a community; thick lines represent the modeled mean prevalence of parasitemia in each trial arm over time. B, Dots depict the estimated mean difference in parasitemia prevalence in percentage points between the 2 arms at each study visit, with 95% CIs. Negative numbers (in the region shaded light blue) indicate a lower prevalence in the azithromycin group. The months during which seasonal malaria chemoprevention (SMC) was administered are shown as gray vertical bars.

As reported previously, the mean prevalence of malaria parasitemia at baseline was 8.9% (95% CI, 5.1%-15.7%) in the azithromycin group and 6.7% (95% CI, 4.0%-12.6%) in the placebo group.^13^ Mean parasitemia estimates were lower in the azithromycin group for all subsequent study visits except for month 48 (Figure 2; eTable 3 in Supplement 1). On average, after adjusting for baseline parasitemia and study time point, the prevalence of parasitemia was lower in the azithromycin group during 4 years of treatment, although the difference was not significantly lower (3.3 percentage points [PP]; 95% CI, −5.8 to −0.2 PP, assuming a 10% prevalence in the placebo arm; permutation *P* = .05; prespecified primary analysis). A sensitivity analysis including the full 5 years of data had similar results (eTable 4 in Supplement 1). Analysis restricted to the period before SMC (ie, months 12, 24, and 36) identified a significantly lower prevalence of parasitemia in the azithromycin arm (4.8 PP lower; 95% CI, −7.4 to −1.3 PP), assuming a 10% prevalence in the placebo arm (permutation *P* = .02).

Parasite density among children with parasitemia was lower in the azithromycin group at each of the postrandomization visits except for month 48 (Table 2), although differences were not significant in the regression model (log-transformed outcome; density, 7040 parasites/μL lower; 95% CI, −11 310 to 430 parasites/μL, assuming a mean parasite density in the placebo arm of 17 000; *P* = .07). No significant differences in gametocyte prevalence or density were observed between the 2 groups (eTable 5 in Supplement 1).

**Table 2.** Individual-Level Parasite Density and Hemoglobin^a^

Month	Parasite density, parasites/μL^b^				Hemoglobin, g/dL			
	Placebo		Azithromycin		Placebo		Azithromycin	
	Children, No.	Mean (95% CI)	No.	Mean (95% CI)	No.	Mean (95% CI)	No.	Mean (95% CI)
0	36	550 (270-1420)	52	70 020 (690-286 200)	542	9.7 (9.6-10.0)	552	9.8 (9.6-10.1)
12	81	22 470 (5660-66 710)	51	400 (260-930)	548	9.1 (8.8-9.5)	551	9.7 (9.0-10.0)
24	28	740 (160-3200)	21	640 (140-1970)	592	10.1 (9.9-10.3)	567	10.2 (9.9-10.3)
36	37	1160 (800-1820)	16	350 (260-480)	571	10.0 (9.8-10.2)	527	10.1 (9.9-10.3)
48	0	NA	5	720 (390-1090)	503	10.2 (10.0-10.3)	459	10.2 (10.0-10.3)
60	112	34 020 (1180-153 040)	70	1180 (440-3680)	518	9.4 (9.3-9.7)	430	9.8 (9.6-10.0)

Abbreviation: NA, not applicable.

SI conversion factors: To convert hemoglobin to grams per liter, multiply by 10.

^a^ Data represent individual-level means with bootstrapped 95% CIs resampled at the community level.

^b^ Among children with malaria parasitemia (rounded to nearest 10).

Mean hemoglobin estimates were higher in the azithromycin arm at all visits except month 48. After adjusting for the mean hemoglobin level at baseline in each community, the hemoglobin concentration was not significantly different between the 2 arms across the first 4 years of treatment (mean of 0.1 g/dL higher in the azithromycin group; 95% CI, −0.1 to 0.2 g/dL; *P* = .44). The prevalence of anemia (hemoglobin <11 g/dL) was 75% (95% CI, 68%-79%) in the azithromycin arm and 78% (95% CI, 73%-83%) in the placebo arm at baseline. The prevalence of anemia during the next 4 years was not significantly lower in the azithromycin arm after adjusting for baseline (square-root-transformed outcome; 4.0 PP lower; 95% CI, −8.8 to 0.9 PP, assuming a 75% prevalence of anemia; *P* = .12) (eTable 6 in Supplement 1). Sensitivity analyses at the 3-year and 5-year end points yielded similar results (eTable 4 in Supplement 1).

## Discussion

A previous study describing 30 communities in Niger found that 2 years of biannual mass azithromycin distributions were associated with a 5.3 PP lower prevalence of malaria parasitemia in children aged 1 to 59 months when the prevalence was 10% in the placebo arm.^13^ All communities continued to receive their originally allocated treatment for an additional 3 years. In the current analysis, we found that the azithromycin arm had a similarly lower prevalence of malaria parasitemia during the third year of the study (ie, 4.8 PP lower than placebo), but the association was not present during the fourth and fifth years, at which time communities were also receiving SMC from the Ministry of Health. Differences between the 2 treatment arms were no longer statistically significant once the SMC program was started.

Azithromycin is a slow-acting antimalarial that inhibits protein translation in the malarial apicoplast.^19^ Azithromycin improves the treatment efficacy of faster-acting antimalarials and is effective for preventing malaria, although less so than alternatives such as doxycycline.^20,21,22,23^ Studies that have randomized individual children to single-dose azithromycin or placebo have failed to demonstrate that azithromycin is effective for preventing malaria parasitemia.^24,25^ However, azithromycin may have a larger effect when given as an MDA because mass azithromycin distributions would in theory provide both a direct preventive effect for a child as well as an indirect effect for other community members by reducing the community load and transmission of parasites. Several cluster randomized trials of azithromycin MDA for trachoma have found reductions in malaria parasitemia.^12,26,27^ The effect of azithromycin on malaria is likely context dependent, however, because other studies of MDA for trachoma have found no effect.^28,29^

The administration of SMC, which was instituted in all study communities after the 36-month time point, may have obscured the effect of azithromycin.^7^ SMC coverage in the study districts was high, ranging from 74% to 95% at each distribution.^30,31^ Randomized trials have found that SMC is effective in reducing malaria and anemia.^32^ Thus, it is likely that the SMC administered during the study reduced the burden of malaria in both the azithromycin and placebo communities at the 48-month and 60-month malaria assessments, which may have contributed to the null outcome at these time points. As such, these results are consistent with a trial conducted in Burkina Faso and Mali that found that children treated with SMC plus a 3-day course of azithromycin had fewer malaria episodes in the 2 weeks after treatment compared with children treated with SMC plus placebo but experienced no difference in malaria incidence throughout the study.^7,33^ Of course, it is also possible that biannual mass azithromycin distributions did not have a clinically meaningful effect on malaria and that the small reductions in malaria parasitemia seen in the early years of the study were simply due to chance, especially given the lower magnitude of effect seen at 24 and 36 months (Figure 2).

For unclear reasons, the prevalence of parasitemia observed at the month 60 visit was higher than that at the other study visits. This finding may simply represent expected seasonal variation, consistent with malaria surveillance published by the Niger Ministry of Health (eTable 7 in Supplement 1).^34^ The month 60 visit took place closer to the preceding malaria season than the other visits (eTable 2 in Supplement 1), so it is possible that children were more likely to have parasitemia left over from the preceding transmission season.^35^ Moreover, parasitemia was assessed at different laboratories throughout the study, and slight variations in laboratory procedures or personnel may have increased measurement variability across the study visits.

### Limitations

This study had limitations. An entire azithromycin-treated community declined to participate after month 36, reducing statistical power and potentially biasing the outcome if this decision was associated with malaria. Individual-level data on SMC were not available, limiting the ability to perform nonprespecified exploratory analyses of the interaction between azithromycin and SMC in individual children. Typically, MDAs were performed outside the malaria transmission season, with the most recent treatment before assessment of malaria outcomes given in the dry season. Although mathematical models have found that this may be a good time of year to reduce the community burden of malaria, the optimal timing is not known, and it is conceivable that treatments during a time of low malaria transmission would reduce the magnitude of any effect.^36^ Due to resource limitations and logistical reasons malaria monitoring was performed only once per year before the transmission season. Additional monitoring visits during and after the rainy season would have been informative. The intervention was administered only to young children given the focus of the main trial on mortality in those younger than 5 years. School-aged children are thought to be a major source of malaria transmission, so targeting preschool-aged children may have limited the impact of any indirect effect of azithromycin on malaria.^37,38^ No data on clinical malaria episodes were collected. The prespecified parasite density outcome may have been especially subject to fluctuations based on the time between infection and sample collection. Numerous exploratory analyses were performed, potentially inflating type I error. Additionally, given geographic heterogeneity in malaria transmission, the results may not be generalizable to areas outside the Sahel and even outside this specific study area.

## Conclusions

In this placebo-controlled cluster randomized trial, communities in which children aged 1 to 59 months were given biannual single-dose mass azithromycin had less malaria than placebo-treated communities during the first 3 years of intervention. However, there was not a significant association between mass azithromycin and malaria parasitemia in the full 5 years of the study, perhaps because its impact was reduced by SMC administered by the Niger Ministry of Health during the final 2 years of the study.

## Notes

Supplement 1. **eTable 1.** Baseline characteristics of the community that declined examinations after month 24 **eTable 2.** Relationship between malaria transmission season, treatment, and examination **eTable 3.** Community-specific prevalence of malaria parasitemia **eTable 4.** Non-pre-specified analyses including all 5 annual study visits after randomization **eTable 5.** Community-specific prevalence of gametocytemia **eTable 6.** Community-specific prevalence of anemia, defined as hemoglobin less than 11 g/dL **eTable 7.** Malaria surveillance reported by the Niger Ministry of Health **eFigure 1.** Map of study area **eFigure 2.** Timing of examination visits and treatment

Supplement 2.
Trial Protocol and Statistical Analysis Plan

Supplement 3.
Members of the MORDOR-Niger Study Group

Supplement 4.
Data Sharing Statement
