# Supplementary material for: Biannual Mass Azithromycin Distributions for Preschool Children and Malaria Parasitemia: A Secondary Analysis of the MORDOR Cluster Randomized Trial
Source: JAMA Netw Open. 2025 Aug 18;8(8):e2527148. doi: 10.1001/jamanetworkopen.2025.27148 (PMC12362227; doi:10.1001/jamanetworkopen.2025.27148)
Supplement: Supplement 1. — eTable 1. Baseline characteristics of the community that declined examinations after month 24 eTable 2. Relationship between malaria transmission season, treatment, and examination eTable 3. Community-specific prevalence of malaria parasitemia eTable 4. Non-pre-specified analyses including all 5 annual study visits after randomization eTable 5. Community-specific prevalence of gametocytemia eTable 6. Community-specific prevalence of anemia, defined as hemoglobin less than 11 g/dL eTable 7. Malaria surveillance reported by the Niger Ministry of Health eFigure 1. Map of study area eFigure 2. Timing of examination visits and treatment [file jamanetwopen-e2527148-s001.pdf]

# Supplemental Online Content

Arzika AM, Abdou A, Maliki R, et al; MORDOR-Niger Study Group. Biannual mass azithromycin distributions for preschool children and malaria parasitemia: a secondary analysis of the MORDOR cluster randomized trial. *JAMA Netw Open.* 2025;8(8):e2527148. doi:10.1001/jamanetworkopen.2025.27148

**eTable 1.** Baseline characteristics of the community that declined examinations after month 24

**eTable 2.** Relationship between malaria transmission season, treatment, and examination

**eTable 3.** Community-specific prevalence of malaria parasitemia

**eTable 4.** Non-pre-specified analyses including all 5 annual study visits after randomization

**eTable 5.** Community-specific prevalence of gametocytemia

**eTable 6.** Community-specific prevalence of anemia, defined as hemoglobin less than 11 g/dL

**eTable 7.** Malaria surveillance reported by the Niger Ministry of Health

**eFigure 1.** Map of study area

**eFigure 2.** Timing of examination visits and treatment

This supplemental material has been provided by the authors to give readers additional information about their work.

© 2025 Arzika AM et al. *JAMA Network Open*.

**eTable 1. Baseline characteristics of the community that declined examinations after month 24.**

| Characteristic | Value |
|---|---|
| Children 1–59 mos, No. | 113 |
| Female, % | 46.9% |
| Age, % | |
| 0y | 12.3% |
| 1y | 14.1% |
| 2y | 18.9% |
| 3y | 17.7% |
| 4y | 37.2% |
| Female head of household, % | 0.9% |
| Number of children 1–59 mos per household, mean (SD) | 3.2 (2.1) |
| Elevation in center of community, m | 185 |
| Distance from center of community to main road, km | 8.7 |
| Distance from center of community to town, km | 19.4 |



© 2025 Arzika AM et al. *JAMA Network Open*.

**eTable 2. Relationship between malaria transmission season, treatment, and examination.** The malaria transmission season was assumed to be July 1 to October 31 each year based on the months of seasonal malaria chemoprevention. The median dates of treatment and examination were used for each study community, and summarized for the treatment arm and monitoring visit as a mean (standard deviation).

| Characteristic | Placebo | Azithromycin |
|---|---|---|
| 2015 monitoring (baseline) | | |
| Month of baseline monitoring visit | Mar 2015 | Mar 2015 |
| Duration between monitoring visit and end of most recent transmission season, months | 4.9 (0.5) | 4.8 (0.4) |
| 2016 monitoring (month 12) | | |
| Month of baseline azithromycin | Jun 2015 | Jun 2015 |
| Month of 6-month azithromycin | Dec 2015 | Dec 2015 |
| Month of 12-month monitoring visit | Jul 2016 | Jun 2016 |
| Duration between monitoring visit and end of most recent transmission season, months | 8.3 (0.6) | 7.3 (1.3) |
| Duration between monitoring visit and most recent azithromycin treatment, months | 6.8 (0.8) | 5.7 (1.5) |
| Duration between second-most recent azithromycin and start of previous transmission season, months | 0.5 (1.4) | 0.3 (1.1) |
| 2017 monitoring (month 24) | | |
| Month of 12-month azithromycin | Jul 2016 | Jul 2016 |
| Month of 18-month azithromycin | Dec 2016 | Dec 2016 |
| Month of 24-month monitoring visit | Apr 2017 | Apr 2017 |
| Duration between monitoring visit and end of most recent transmission season | 5.8 (0.9) | 5.6 (0.9) |
| Duration between monitoring visit and most recent azithromycin treatment | 4.7 (0.8) | 4.4 (1.0) |
| Duration between second-most recent azithromycin and start of previous transmission season, months | -0.6 (0.4) | -0.1 (0.6) |
| 2018 monitoring (month 36) | | |
| Month of 24-month azithromycin | May 2017 | May 2017 |
| Month of 30-month azithromycin | Feb 2018 | Feb 2018 |
| Month of 36-month monitoring visit | May 2018 | May 2018 |
| Duration between monitoring visit and end of most recent transmission season, months | 7.0 (0.6) | 6.8 (0.4) |
| Duration between monitoring visit and most recent azithromycin treatment, months | 3.7 (0.4) | 3.6 (0.4) |
| Duration between second-most recent azithromycin and start of previous transmission season, months | 1.4 (0.5) | 1.5 (0.4) |
| 2019 monitoring (month 48) | | |
| Month of 36-month azithromycin | Jun 2018 | Jun 2018 |
| Month of 42-month azithromycin | Dec 2018 | Dec 2018 |
| Month of 48-month monitoring visit | May 2019 | Apr 2019 |
| Duration between monitoring visit and end of most recent transmission season, months | 6.2 (0.6) | 5.9 (0.5) |
| Duration between monitoring visit and most recent azithromycin treatment, months | 4.7 (0.5) | 4.3 (0.6) |
| Duration between second-most recent azithromycin and start of previous transmission season, months | 0.1 (0.1) | 0.04 (0.2) |
| 2020 monitoring (month 60) | | |
| Month of 48-month azithromycin | May 2019 | May 2019 |
| Month of 54-month azithromycin | Nov 2019 | Nov 2019 |
| Month of 60-month monitoring visit | Feb 2020 | Feb 2020 |
| Duration between monitoring visit and end of most recent transmission season, months | 3.6 (0.3) | 3.5 (0.3) |
| Duration between monitoring visit and most recent azithromycin treatment, months | 3.5 (0.6) | 3.8 (1.3) |
| Duration between second-most recent azithromycin and start of previous transmission season, months | 1.0 (0.7) | 1.6 (1.4) |



© 2025 Arzika AM et al. *JAMA Network Open.*

**eTable 3. Community-specific prevalence of malaria parasitemia**

| Community | Baseline population | No. positive / total tested, per community (%) | | | | | |
|---|---|---|---|---|---|---|---|
| | | Month 0 | Month 12 | Month 24 | Month 36 | Month 48 | Month 60 |
| Placebo | | | | | | | |
| 1 | 398 | 0/40 (0%) | 5/40 (13%) | 4/40 (10%) | 3/41 (7%) | 0/35 (0%) | 11/31 (35%) |
| 2 | 38 | 2/23 (9%) | 4/28 (14%) | 2/31 (7%) | 2/27 (7%) | 0/29 (0%) | 2/11 (18%) |
| 3 | 232 | 0/38 (0%) | 6/28 (21%) | 0/42 (0%) | 0/40 (0%) | 0/37 (0%) | 4/40 (10%) |
| 4 | 302 | 2/42 (5%) | 2/41 (5%) | 0/41 (0%) | 1/42 (2%) | 0/36 (0%) | 5/40 (12%) |
| 5 | 335 | 11/37 (30%) | 9/37 (24%) | 3/41 (7%) | 1/42 (2%) | 0/40 (0%) | 12/40 (30%) |
| 6 | 146 | 4/37 (11%) | 3/36 (8%) | 1/42 (2%) | 3/40 (7%) | 0/36 (0%) | 1/40 (3%) |
| 7 | 27 | 1/22 (5%) | 5/19 (26%) | 1/25 (4%) | 6/32 (19%) | 0/34 (0%) | 7/37 (19%) |
| 8 | 131 | 1/29 (3%) | 8/39 (21%) | 3/42 (7%) | 1/41 (2%) | 0/35 (0%) | 14/39 (36%) |
| 9 | 278 | 1/43 (2%) | 2/38 (5%) | 1/44 (2%) | 0/44 (0%) | 0/34 (0%) | 13/40 (32%) |
| 10 | 110 | 4/36 (11%) | 3/40 (8%) | 1/40 (3%) | 4/38 (11%) | 0/23 (0%) | 4/13 (31%) |
| 11 | 140 | 2/40 (5%) | 4/40 (10%) | 3/41 (7%) | 3/40 (7%) | 0/28 (0%) | 10/41 (24%) |
| 12 | 198 | 0/37 (0%) | 2/40 (5%) | 1/41 (2%) | 0/40 (0%) | 0/40 (0%) | 1/26 (4%) |
| 13 | 323 | 4/41 (10%) | 14/40 (35%) | 2/41 (5%) | 3/44 (7%) | 0/38 (0%) | 8/39 (21%) |
| 14 | 314 | 4/39 (10%) | 12/41 (29%) | 4/41 (10%) | 10/40 (25%) | 0/37 (0%) | 15/41 (37%) |
| 15 | 59 | 0/38 (0%) | 2/41 (5%) | 2/40 (5%) | 0/20 (0%) | 0/23 (0%) | 5/40 (12%) |
| *Mean (95%CI)* | | *7% (4-13%)* | *15% (11-21%)* | *5% (3-6%)* | *7% (4-11%)* | *0% (0-1%)* | *22% (16-27%)* |
| Azithromycin | | | | | | | |
| 16 | 58 | 4/40 (10%) | 2/42 (5%) | 4/42 (10%) | 0/42 (0%) | 0/28 (0%) | 8/38 (21%) |
| 17 | 32 | 1/24 (4%) | 9/39 (23%) | 2/33 (6%) | 0/29 (0%) | 5/22 (23%) | 11/18 (61%) |
| 18 | 139 | 1/40 (3%) | 11/38 (29%) | 2/40 (5%) | 1/40 (3%) | 0/36 (0%) | 7/25 (28%) |
| 19 | 39 | 3/32 (9%) | 0/28 (0%) | 0/21 (0%) | 2/24 (8%) | 0/21 (0%) | 5/13 (38%) |
| 20 | 93 | 15/39 (39%) | 2/39 (5%) | 0/41 (0%) | 2/45 (4%) | 0/32 (0%) | 7/23 (30%) |
| 21 | 58 | 1/30 (3%) | 0/42 (0%) | 1/40 (3%) | 0/40 (0%) | 0/36 (0%) | 1/40 (3%) |
| 22 | 376 | 0/40 (0%) | 4/40 (10%) | 4/40 (10%) | 2/39 (5%) | 0/33 (0%) | 12/39 (31%) |
| 23 | 212 | 0/39 (0%) | 3/39 (8%) | 0/40 (0%) | 1/40 (3%) | 0/36 (0%) | 1/41 (2%) |
| 24 | 127 | 0/41 (0%) | 4/41 (10%) | 3/42 (7%) | 0/44 (0%) | 0/39 (0%) | 1/32 (3%) |
| 25 | 85 | 7/39 (18%) | 8/38 (21%) | 0/40 (0%) | 4/42 (10%) | 0/37 (0%) | 1/34 (3%) |
| 26 | 41 | 0/26 (0%) | 0/16 (0%) | 1/40 (3%) | 1/35 (3%) | 0/41 (0%) | 1/40 (3%) |
| 27 | 172 | 8/46 (17%) | 1/39 (5%) | 3/42 (7%) | 1/42 (2%) | 0/42 (0%) | 5/39 (13%) |
| 28 | 49 | 2/37 (5%) | 1/32 (3%) | 0/23 (0%) | 0/25 (0%) | 0/20 (0%) | 1/12 (8%) |
| 29 | 101 | 5/38 (13%) | 1/40 (3%) | 1/43 (2%) | 2/40 (5%) | 0/38 (0%) | 9/36 (25%) |
| 30 | 113 | 5/41 (12%) | 5/38 (13%) | 0/40 (0%) | 0/0 | 0/0 | 0/0 |
| *Mean (95%CI)* | | *9% (5-16%)* | *9% (5-14%)* | *3% (2-5%)* | *3% (2-5%)* | *2% (0-5%)* | *19% (12-30%)* |

Annual monitoring visits were planned for a random sample of 40 children per community, with a separate random sample at each visit. In practice, 50 children per community were invited under the assumption that approximately 20% would not attend the visit. Thus the number of children sampled per community sometimes exceeded 40. The study team returned to study communities multiple times to improve coverage. When data were collected on fewer than 40 children per community this was usually due to the community having a relatively small number of children eligible for monitoring or due to seasonal migration.



© 2025 Arzika AM et al. *JAMA Network Open*.

**eTable 4. Non-pre-specified analyses including all 5 annual study visits after randomization.** For outcomes that were square-root-transformed (prevalence of malaria parasitemia, prevalence of anemia, and parasite density) the magnitude of the association is expressed assuming the specified value in the placebo group.

| | Placebo group assumption | Estimated magnitude of effect relative to assumed value in placebo group | Permutation P-value |
|---|---|---|---|
| **Month 36 (before SMC started)** | | | |
| Community-level outcomes | | | |
| Prevalence of parasitemia | 10% | 4.8 pp lower in azithromycin arm (95%CI -7.4 to -1.3) | 0.02 |
| Prevalence of anemia | 75% | 4.8 pp lower in azithromycin arm (95%CI -10.0 to 0.5) | 0.09 |
| Individual-level outcomes | | | |
| Parasite density | 17,000 | 7050 parasites/μl lower in azithromycin arm (95%CI -11330 to 450) | 0.07 |
| Hemoglobin | NA | 0.1 g/dl higher in azithromycin arm (-0.1 to 0.4) | 0.26 |
| **Month 60 (final study visit)** | | | |
| Community-level outcomes | | | |
| Prevalence of parasitemia | 10% | 3.4 pp lower in azithromycin arm (95%CI -6.1 to 0.1) | 0.07 |
| Prevalence of anemia | 75% | 3.7 pp lower in azithromycin arm (95%CI -8.0 to 0.8) | 0.11 |
| Individual-level outcomes | | | |
| Parasite density | 17,000 | 6920 parasites/μl lower in azithromycin arm (95%CI -11070 to 120) | 0.09 |
| Hemoglobin | NA | 0.2 g/dl higher in azithromycin arm (0.02 to 0.3) | 0.06 |

pp = percentage points; SMC = seasonal malaria chemoprevention



© 2025 Arzika AM et al. *JAMA Network Open.*

**eTable 5. Community-specific prevalence of gametocytemia.**

| Community | No. positive / total tested, per community (%) | | | | | |
|---|---|---|---|---|---|---|
| | Month 0 | Month 12 | Month 24 | Month 36 | Month 48 | Month 60 |
| Placebo[a] | | | | | | |
| 1 | 0/40 (0%) | 0/40 (0%) | 0/40 (0%) | 0/41 (0%) | 0/35 (0%) | 0/31 (0%) |
| 2 | 0/23 (0%) | 0/28 (0%) | 0/31 (0%) | 0/27 (0%) | 0/29 (0%) | 0/11 (0%) |
| 3 | 0/38 (0%) | 0/28 (0%) | 0/42 (0%) | 0/40 (0%) | 0/37 (0%) | 1/40 (3%) |
| 4 | 0/42 (0%) | 0/41 (0%) | 0/41 (0%) | 0/42 (0%) | 0/36 (0%) | 0/40 (0%) |
| 5 | 2/37 (5%) | 1/37 (3%) | 0/41 (0%) | 0/42 (0%) | 0/40 (0%) | 0/40 (0%) |
| 6 | 0/37 (0%) | 0/36 (0%) | 0/42 (0%) | 0/40 (0%) | 0/36 (0%) | 0/40 (0%) |
| 7 | 0/22 (0%) | 0/19 (0%) | 0/25 (0%) | 0/32 (0%) | 0/34 (0%) | 0/37 (0%) |
| 8 | 0/29 (0%) | 0/39 (0%) | 0/42 (0%) | 0/41 (0%) | 0/35 (0%) | 1/39 (3%) |
| 9 | 0/43 (0%) | 0/38 (0%) | 0/44 (0%) | 0/44 (0%) | 0/34 (0%) | 0/40 (0%) |
| 10 | 0/36 (0%) | 0/40 (0%) | 0/40 (0%) | 0/38 (0%) | 0/23 (0%) | 0/13 (0%) |
| 11 | 0/40 (0%) | 0/40 (0%) | 1/41 (2%) | 0/40 (0%) | 0/28 (0%) | 0/41 (0%) |
| 12 | 0/37 (0%) | 0/40 (0%) | 0/41 (0%) | 0/40 (0%) | 0/40 (0%) | 0/26 (0%) |
| 13 | 0/41 (0%) | 0/40 (0%) | 0/41 (0%) | 0/44 (0%) | 0/38 (0%) | 0/39 (0%) |
| 14 | 0/39 (0%) | 1/41 (2%) | 1/41 (2%) | 0/40 (0%) | 0/37 (0%) | 0/41 (0%) |
| 15 | 0/38 (0%) | 0/41 (0%) | 0/40 (0%) | 0/20 (0%) | 0/23 (0%) | 0/40 (0%) |
| *Mean (95%CI)* | *0% (0-1%)* | *0% (0-1%)* | *0% (0-1%)* | *0% (0-1%)* | *0% (0-1%)* | *0% (0-1%)* |
| Azithromycin[b] | | | | | | |
| 16 | 0/40 (0%) | 0/42 (0%) | 0/42 (0%) | 0/42 (0%) | 0/28 (0%) | 0/38 (0%) |
| 17 | 0/24 (0%) | 0/39 (0%) | 0/33 (0%) | 0/29 (0%) | 0/22 (0%) | 0/18 (0%) |
| 18 | 1/40 (3%) | 0/38 (0%) | 0/40 (0%) | 0/40 (0%) | 0/36 (0%) | 0/25 (0%) |
| 19 | 0/32 (0%) | 0/28 (0%) | 0/21 (0%) | 0/24 (0%) | 0/21 (0%) | 0/13 (0%) |
| 20 | 0/39 (0%) | 0/39 (0%) | 0/41 (0%) | 0/45 (0%) | 0/32 (0%) | 0/23 (0%) |
| 21 | 0/30 (0%) | 0/42 (0%) | 0/40 (0%) | 0/40 (0%) | 0/36 (0%) | 0/40 (0%) |
| 22 | 0/40 (0%) | 0/40 (0%) | 1/40 (3%) | 0/39 (0%) | 0/33 (0%) | 0/39 (0%) |
| 23 | 0/39 (0%) | 0/39 (0%) | 0/40 (0%) | 0/40 (0%) | 0/36 (0%) | 0/41 (0%) |
| 24 | 0/41 (0%) | 0/41 (0%) | 0/42 (0%) | 0/44 (0%) | 0/39 (0%) | 0/32 (0%) |
| 25 | 0/39 (0%) | 0/38 (0%) | 0/40 (0%) | 0/42 (0%) | 0/37 (0%) | 0/34 (0%) |
| 26 | 0/26 (0%) | 0/16 (0%) | 0/40 (0%) | 0/35 (0%) | 0/41 (0%) | 0/40 (0%) |
| 27 | 0/46 (0%) | 0/39 (0%) | 0/42 (0%) | 0/42 (0%) | 0/42 (0%) | 0/39 (0%) |
| 28 | 0/37 (0%) | 0/32 (0%) | 0/23 (0%) | 0/25 (0%) | 0/20 (0%) | 0/12 (0%) |
| 29 | 0/38 (0%) | 0/40 (0%) | 0/43 (0%) | 0/40 (0%) | 0/38 (0%) | 0/36 (0%) |
| 30 | 0/41 (0%) | 0/38 (0%) | 0/40 (0%) | 0/0 | 0/0 | 0/0 |
| *Mean (95%CI)* | *0% (0-1%)* | *0% (0-1%)* | *0% (0-1%)* | *0% (0-1%)* | *0% (0-1%)* | *0% (0-1%)* |

[a] Gametocyte densities in placebo arm: Month 0: 60, 130; Month 12: 10, 100; Month 24: 10, 10; Month 60: 10,10.
[b] Gametocyte densities in azithromycin arm: Month 0: 400 Month 24: 10



© 2025 Arzika AM et al. *JAMA Network Open*.

**eTable 6. Community-specific prevalence of anemia, defined as hemoglobin less than 11 g/dL**

| | No. positive / total tested, per community (%) | | | | | |
|---|---|---|---|---|---|---|
| Community | Month 0 | Month 12 | Month 24 | Month 36 | Month 48 | Month 60 |
| Placebo | | | | | | |
| 1 | 33/40 (82%) | 36/40 (90%) | 27/40 (68%) | 31/41 (76%) | 24/35 (69%) | 25/31 (81%) |
| 2 | 16/23 (70%) | 23/28 (82%) | 19/31 (61%) | 23/27 (85%) | 25/29 (86%) | 6/11 (55%) |
| 3 | 27/38 (71%) | 26/28 (93%) | 29/42 (69%) | 34/40 (85%) | 25/36 (69%) | 34/40 (85%) |
| 4 | 28/42 (67%) | 37/41 (90%) | 22/41 (54%) | 24/42 (57%) | 24/36 (67%) | 31/40 (78%) |
| 5 | 26/37 (70%) | 37/37 (100%) | 29/41 (71%) | 26/42 (62%) | 29/40 (72%) | 37/40 (92%) |
| 6 | 32/37 (86%) | 31/36 (86%) | 24/42 (57%) | 24/40 (60%) | 26/36 (72%) | 33/40 (82%) |
| 7 | 20/22 (91%) | 16/19 (84%) | 17/25 (68%) | 29/32 (91%) | 22/34 (65%) | 32/37 (86%) |
| 8 | 27/29 (93%) | 31/39 (79%) | 28/42 (67%) | 31/41 (76%) | 22/35 (63%) | 33/39 (85%) |
| 9 | 37/43 (86%) | 21/38 (55%) | 28/44 (64%) | 31/44 (70%) | 27/34 (79%) | 33/40 (82%) |
| 10 | 26/36 (72%) | 37/40 (92%) | 36/40 (90%) | 26/38 (68%) | 20/22 (91%) | 10/13 (77%) |
| 11 | 26/40 (65%) | 31/40 (78%) | 31/41 (76%) | 26/40 (65%) | 15/28 (54%) | 28/41 (68%) |
| 12 | 25/37 (68%) | 35/40 (88%) | 28/41 (68%) | 30/40 (75%) | 28/40 (70%) | 16/26 (62%) |
| 13 | 36/41 (88%) | 36/40 (90%) | 31/41 (76%) | 41/44 (93%) | 24/38 (63%) | 33/39 (85%) |
| 14 | 33/39 (85%) | 34/41 (83%) | 29/41 (71%) | 37/40 (92%) | 32/37 (86%) | 38/41 (93%) |
| 15 | 28/38 (74%) | 34/41 (83%) | 32/40 (80%) | 14/20 (70%) | 19/23 (83%) | 33/40 (82%) |
| *Mean (95%CI)* | *78% (73-83%)* | *85% (78-89%)* | *69% (65-74%)* | *75% (70-81%)* | *73% (68-78%)* | *80% (73-84%)* |
| Azithromycin | | | | | | |
| 16 | 35/40 (88%) | 26/42 (62%) | 29/42 (69%) | 38/42 (90%) | 22/28 (79%) | 29/38 (76%) |
| 17 | 18/24 (75%) | 25/39 (64%) | 21/33 (64%) | 21/29 (72%) | 13/21 (62%) | 14/18 (78%) |
| 18 | 28/40 (70%) | 33/38 (87%) | 28/40 (70%) | 27/40 (68%) | 27/36 (75%) | 22/25 (88%) |
| 19 | 28/32 (88%) | 16/28 (57%) | 19/21 (90%) | 17/24 (71%) | 17/21 (81%) | 10/13 (77%) |
| 20 | 33/39 (85%) | 33/39 (85%) | 30/41 (73%) | 33/45 (73%) | 21/32 (66%) | 19/23 (83%) |
| 21 | 21/30 (70%) | 28/42 (67%) | 20/40 (50%) | 23/40 (58%) | 24/36 (67%) | 28/40 (70%) |
| 22 | 30/40 (75%) | 28/40 (70%) | 28/40 (70%) | 27/39 (69%) | 22/33 (67%) | 33/39 (85%) |
| 23 | 30/39 (77%) | 39/39 (100%) | 31/40 (78%) | 30/40 (75%) | 27/36 (75%) | 29/41 (71%) |
| 24 | 27/41 (66%) | 21/41 (51%) | 29/42 (69%) | 30/44 (68%) | 24/39 (62%) | 19/32 (59%) |
| 25 | 31/39 (79%) | 33/38 (87%) | 30/40 (75%) | 33/42 (79%) | 29/37 (78%) | 27/34 (79%) |
| 26 | 21/26 (81%) | 10/16 (62%) | 32/40 (80%) | 23/35 (66%) | 28/41 (68%) | 26/40 (65%) |
| 27 | 35/46 (76%) | 26/39 (67%) | 28/42 (67%) | 33/42 (79%) | 34/42 (81%) | 34/39 (87%) |
| 28 | 17/37 (46%) | 16/32 (50%) | 11/23 (48%) | 12/25 (48%) | 10/20 (50%) | 7/12 (58%) |
| 29 | 28/38 (74%) | 31/40 (78%) | 33/43 (77%) | 32/40 (80%) | 27/37 (73%) | 30/36 (83%) |
| 30 | 29/41 (71%) | 29/38 (76%) | 24/40 (60%) | 0/0 | 0/0 | 0/0 |
| *Mean (95%CI)* | *75% (68-79%)* | *71% (64-78%)* | *69% (64-74%)* | *71% (65-76%)* | *70% (65-74%)* | *76% (70-80%)* |



© 2025 Arzika AM et al. *JAMA Network Open.*

**eTable 7. Malaria surveillance reported by the Niger Ministry of Health.** Values indicate the number of malaria cases among 1–4 year-olds in the Dosso region, as reported in the table of 10 principal Illnesses in the annual statistical report (https://www.stat-niger.org/?page_id=500; accessed April 25, 2025).

| | Malaria cases, 1–4 year-olds | |
|---|---|---|
| Year | Dosso Region | Niger (nationwide) |
| 2015 | 291,191 | 1,653,908 |
| 2016 | 283,578 | 1,551,419 |
| 2017 | 79,247 | 1,385,056 |
| 2018 | 226,760 | 1,536,159 |
| 2019 | 264,344 | 1,621,751 |



© 2025 Arzika AM et al. *JAMA Network Open*.

**eFigure 1. Map of study area.** The trial was done in the departments of Loga and Boboye, Dosso Region, Niger. The darker points represent the 30 study communities in the ancillary trial (i.e., the present study); the lighter points represent the communities in the main trial. The black dots indicate the department seats. Lines represent roads.

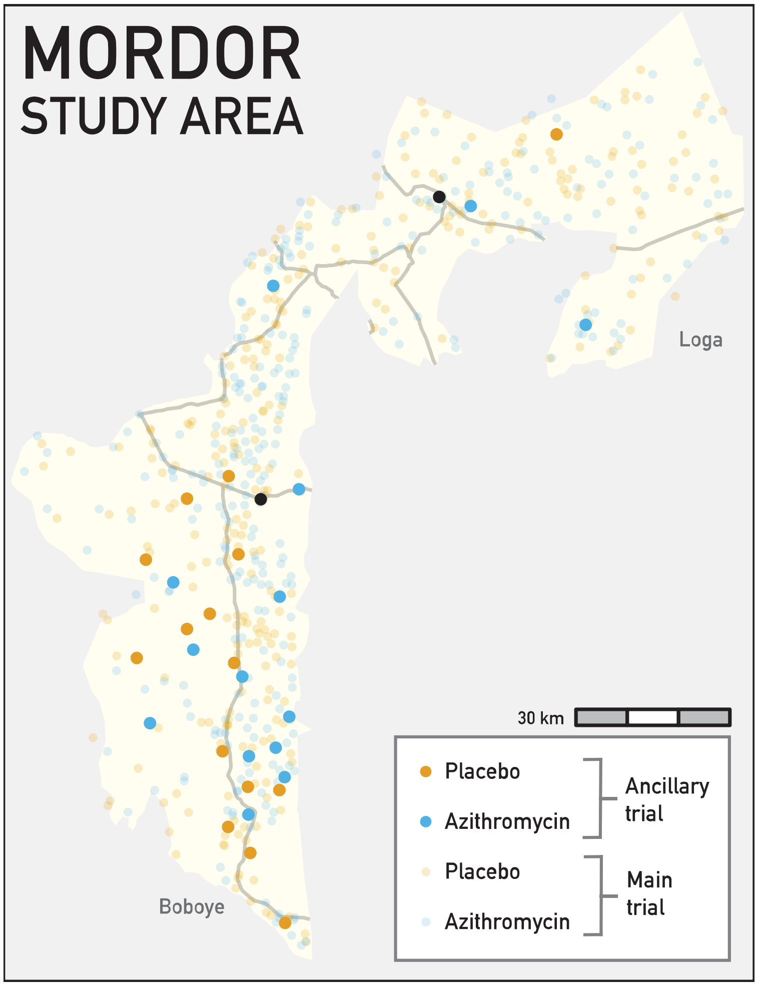




© 2025 Arzika AM et al. *JAMA Network Open.*

**eFigure 2. Timing of examination visits and treatment.** The timing of examinations (black diamond markers) and treatments (blue oval markers for azithromycin; orange oval markers for placebo) is depicted over the 5-year study period. Time is shown on the x-axis; each community is a row of the y-axis. Markers indicate the median date of the examination or treatment for each community. The shading of the gray bars represents the monthly precipitation in the study area, scaled from 0 to 1 over the study period (https://data.humdata.org/dataset/ner-rainfall-subnational; accessed April 26, 2025).

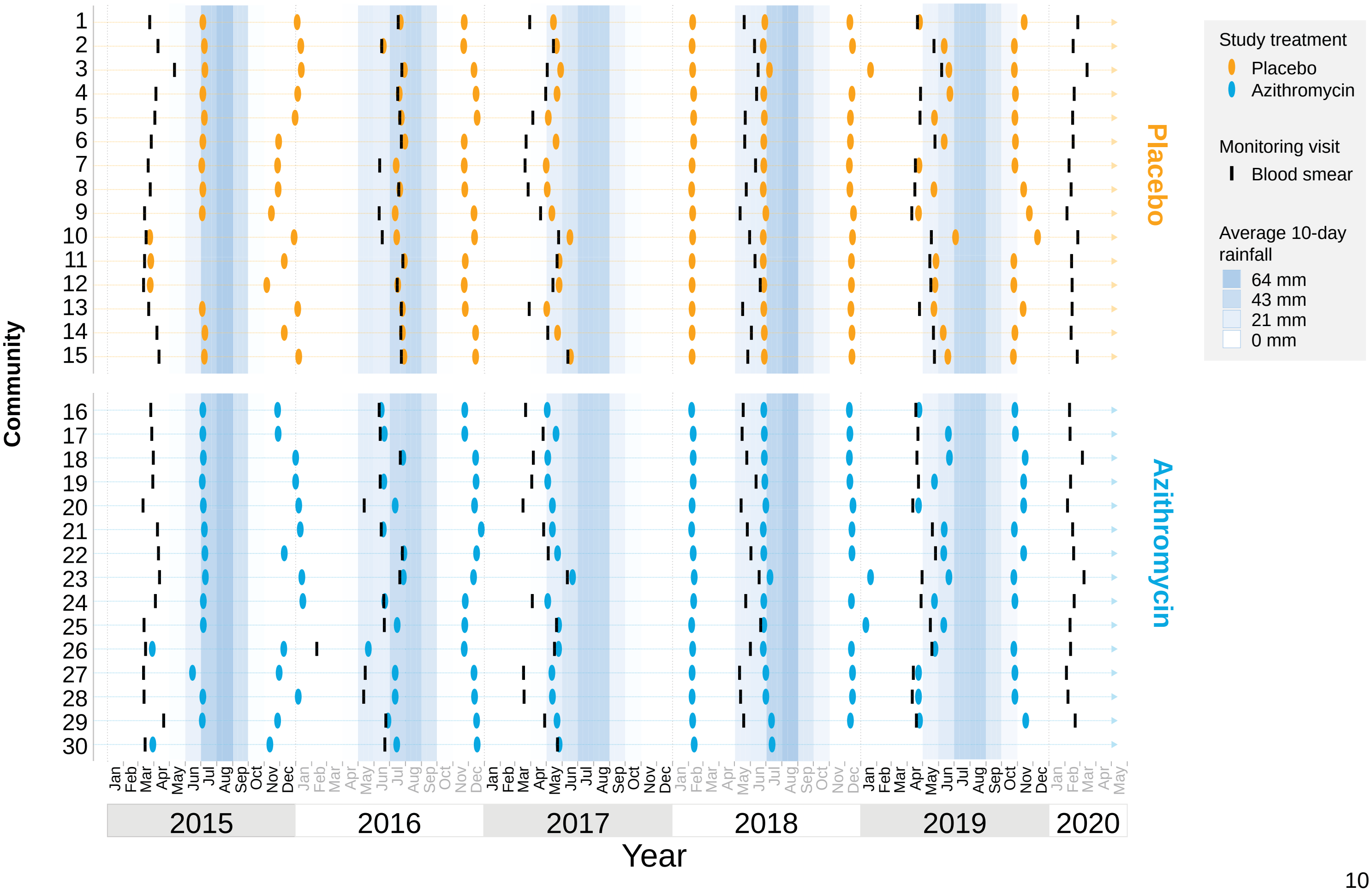




© 2025 Arzika AM et al. *JAMA Network Open.*
