# Supplementary material for: Biannual Mass Azithromycin Distributions for Preschool Children and Malaria Parasitemia: A Secondary Analysis of the MORDOR Cluster Randomized Trial
Source: JAMA Netw Open. 2025 Aug 18;8(8):e2527148. doi: 10.1001/jamanetworkopen.2025.27148 (PMC12362227; doi:10.1001/jamanetworkopen.2025.27148)
Supplement: Supplement 3. — Members of the MORDOR-Niger Study Group [file jamanetwopen-e2527148-s003.pdf]

Supplemental Online Content: Nonauthor Collaborators
*First name, last name, and suffix (if applicable) are required and will appear in PubMed.

| *Group Name(s): MORDOR-Niger Study Group | | | | | | | |
|---|---|---|---|---|---|---|---|
| ***First Name and Middle Initial(s)** | ***Last Name** | ***Suffix (eg, Jr, III)** | Academic Degrees | Institution | Location (city, state/province, country) | Role or Contribution, eg, chair, principal investigator | Group (if more than 1 Group listed in the byline) and/or Subgroup (eg, Steering Committee) |
| Paul M | Emerson | | PhD | International Trachoma Initiative | Decatur, GA | co-investigator | |
| Huub | Gelderblom | | MD, PhD, MPH | International Trachoma Initiative | Decatur, GA | co-investigator | |
| PJ | Hooper | | MA | International Trachoma Initiative | Decatur, GA | co-investigator | |
| Jerusha | Weaver | | MPH | Johns Hopkins University | Baltimore, MD | co-investigator | |
| Sheila K | West | | PhD | Johns Hopkins University | Baltimore, MD | co-investigator, steering committee | |
| Robin L | Bailey | | MA, BM, MRCP, DTM&H, PhD, FRCP | London School of Hygiene and Tropical Medicine | London, UK | co-investigator, steering committee | |
| John | Hart | | MD | London School of Hygiene and Tropical Medicine | London, UK | co-investigator | |
| Amza | Abdou | | MD | Programme National de Santé Oculaire | Niamey, Niger | co-investigator | |
| Nassirou | Beido | | MS | Programme National de Santé Oculaire | Niamey, Niger | co-investigator | |
| Boubacar | Kadri | | MD | Programme National de Santé Oculaire | Niamey, Niger | co-investigator | |
| Maria M | Ali | | BA | The Carter Center | Niamey, Niger | co-investigator | |
| Mankara K | Alio | | MD | The Carter Center | Niamey, Niger | co-investigator | |
| Ahmed | Arzika | | MPH | The Carter Center | Niamey, Niger | co-investigator | |
| Nameywa | Boubacar | | MD | The Carter Center | Niamey, Niger | co-investigator | |
| E Kelly | Callahan | | MPH | The Carter Center | Atlanta, GA | co-investigator | |
| Sanoussi | Elh Adamou | | MD | The Carter Center | Niamey, Niger | co-investigator | |
| Nana Fatima | Galo | | RN | The Carter Center | Niamey, Niger | co-investigator | |
| Fatima | Ibrahim | | RN | The Carter Center | Niamey, Niger | co-investigator | |
| Salissou | Kane | | PhD | The Carter Center | Niamey, Niger | co-investigator | |

Supplemental Online Content: Nonauthor Collaborators
*First name, last name, and suffix (if applicable) are required and will appear in PubMed.

| ***First Name and Middle Initial(s)** | ***Last Name** | ***Suffix (eg, Jr, III)** | Academic Degrees | Institution | Location (city, state/province, country) | Role or Contribution, eg, chair, principal investigator | Group (if more than 1 Group listed in the byline) and/or Subgroup (eg, Steering Committee) |
|---|---|---|---|---|---|---|---|
| Mariama | Kiemago | | RN | The Carter Center | Niamey, Niger | co-investigator | |
| Ramatou | Maliki | | MPH | The Carter Center | Niamey, Niger | co-investigator | |
| Aisha E | Stewart | | MPH | The Carter Center | Atlanta, GA | co-investigator | |
| Cindi | Chen | | MS | University of California, San Francisco | San Francisco, CA | co-investigator | |
| Catherine | Cook | | MPH | University of California, San Francisco | San Francisco, CA | co-investigator | |
| Sun Y | Cotter | | MPH | University of California, San Francisco | San Francisco, CA | co-investigator | |
| Thuy | Doan | | MD, PhD | University of California, San Francisco | San Francisco, CA | co-investigator | |
| Bruce D | Gaynor | | MD | University of California, San Francisco | San Francisco, CA | co-investigator | |
| Armin | Hinterwirth | | PhD | University of California, San Francisco | San Francisco, CA | co-investigator | |
| Jeremy D | Keenan | | MD, MPH | University of California, San Francisco | San Francisco, CA | co-investigator, steering committee | |
| Elodie | Lebas | | RN | University of California, San Francisco | San Francisco, CA | co-investigator | |
| Thomas M | Lietman | | MD | University of California, San Francisco | San Francisco, CA | principal investigator, steering committee | |
| Ying | Lin | | MPH | University of California, San Francisco | San Francisco, CA | co-investigator | |
| Kieran S | O'Brien | | PhD, MPH | University of California, San Francisco | San Francisco, CA | co-investigator | |
| Catherine E | Oldenburg | | ScD, MPH | University of California, San Francisco | San Francisco, CA | co-investigator | |
| Travis C | Porco | | PhD, MPH | University of California, San Francisco | San Francisco, CA | co-investigator, steering committee | |

Supplemental Online Content: Nonauthor Collaborators
*First name, last name, and suffix (if applicable) are required and will appear in PubMed.

| ***First Name and Middle Initial(s)** | ***Last Name** | ***Suffix (eg, Jr, III)** | Academic Degrees | Institution | Location (city, state/province, country) | Role or Contribution, eg, chair, principal investigator | Group (if more than 1 Group listed in the byline) and/or Subgroup (eg, Steering Committee) |
|---|---|---|---|---|---|---|---|
| David A | Ramirez | | MD | University of California, San Francisco | San Francisco, CA | co-investigator | |
| Kathryn J | Ray | | PhD | University of California, San Francisco | San Francisco, CA | co-investigator | |
| Philip J | Rosenthal | | MD | University of California, San Francisco | San Francisco, CA | co-investigator | |
| George W | Rutherford | | MD | University of California, San Francisco | San Francisco, CA | co-investigator | |
| Benjamin | Vanderschelden | | BSc | University of California, San Francisco | San Francisco, CA | co-investigator | |
| Nicole E | Varnado | | MPH | University of California, San Francisco | San Francisco, CA | co-investigator | |
| John P | Whitcher | | MD, MPH | University of California, San Francisco | San Francisco, CA | co-investigator | |
| Dionna M | Wittberg | | MPH | University of California, San Francisco | San Francisco, CA | co-investigator | |
| Lee | Worden | | PhD | University of California, San Francisco | San Francisco, CA | co-investigator | |
| Lina | Zhong | | BS | University of California, San Francisco | San Francisco, CA | co-investigator | |
| Zhaoxia | Zhou | | BS | University of California, San Francisco | San Francisco, CA | co-investigator | |
