# Supplementary material for: Biannual Mass Azithromycin Distributions for Preschool Children and Malaria Parasitemia: A Secondary Analysis of the MORDOR Cluster Randomized Trial
Source: JAMA Netw Open. 2025 Aug 18;8(8):e2527148. doi: 10.1001/jamanetworkopen.2025.27148 (PMC12362227; doi:10.1001/jamanetworkopen.2025.27148)
Supplement: Supplement 4. — Data Sharing Statement [file jamanetwopen-e2527148-s004.pdf]

Data Sharing Statement

Arzika. Biannual Mass Azithromycin Distributions for Preschool Children and Malaria Parasitemia. *JAMA Netw Open*. Published August 18, 2025.
doi:10.1001/jamanetworkopen.2025.27148

Data
Additional Information: ClinicalTrials.gov NCT02048007
Data available: Yes
Data types: Deidentified participant data
How to access data: Repository: https://osf.io/nh8tk
When available: With publication

Supporting Documents
Document types: None

Additional Information
Who can access the data: Anyone
Types of analyses: Any purpose
Mechanisms of data availability: Without investigator support
Any additional restrictions: None
